# Progesterone and Cerebral Function during Emotion Processing in Men and Women with Schizophrenia

**DOI:** 10.1155/2012/917901

**Published:** 2012-02-15

**Authors:** Julie Champagne, Nadia Lakis, Josiane Bourque, Emmanuel Stip, Olivier Lipp, Adrianna Mendrek

**Affiliations:** ^1^Centre de Recherche Fernand-Séguin, Hôpital Louis-Hippolyte Lafontaine, 7331 Rue Hochelaga, Montréal, QC, Canada H1N 3V2; ^2^Department of Psychiatry, Faculty of Medicine, University of Montreal, Montréal, QC, Canada H3C 3J7

## Abstract

Schizophrenia has been associated with disturbed levels of sex-steroid hormones, including estrogen and testosterone. In the present study we have examined the implication of a less studied hormone progesterone. Forty-three patients with schizophrenia (21 women) and 43 control participants (21 women) underwent functional MRI while viewing emotionally positive, negative, and neutral images. Blood samples were taken prior to the scanning session to evaluate progesterone levels. Simple regression analyses between levels of progesterone and brain activations associated with emotion processing were performed using SPM5. A positive correlation was found between progesterone levels and brain activations during processing of emotionally charged images in both healthy and schizophrenia men, but no significant relationship was revealed in women. These preliminary results indicate that progesterone is significantly associated with brain activations during processing of positive and negative affect in healthy and schizophrenia men, but not in women. Further investigation is warranted.

## 1. Introduction

There is some evidence of a relationship between sex-steroid hormones (i.e., estrogen, testosterone, and less commonly progesterone) and emotion processing in the general population [1–3]. Fluctuations in estrogen and progesterone have been linked with increased vulnerability to mood disorders in women, while elevated levels of testosterone have been primarily associated with antisocial behaviours, behaviours of dominance, and aggressiveness in both men and women [4]. In schizophrenia, some studies have found abnormal levels of estrogens and testosterone in patients, but the results have been equivocal and sometimes attributed to the antipsychotic-induced hyperprolactinemia, which may alter levels of gonadal hormones [5]. Despite numerous studies and clinical observations of lower relapse of clinical symptoms during pregnancy, high relapse postpartum, and the fluctuation of symptoms across the menstrual cycle (attributed typically to the changing levels of estrogens), a link between progesterone and affect in schizophrenia has yet to be examined [6].

The little emphasis that has been placed on the relationship between progesterone and emotional functioning has been explored primarily in healthy women because this hormone is a female reproductive hormone. Nonetheless, it is produced in both men and women, and recent evidence suggests that it is implicated in brain function of both sexes. Thus, progesterone has been shown to play an important role in mood regulation [7], cognition [8], inflammation, mitochondrial function, neurogenesis and regeneration, myelination, and recovery from traumatic brain injury in both men and women [9].

The goal of the present study was to explore progesterone's implication in neural correlates of emotion processing in both healthy and schizophrenia men and women.

## 2. Methods

### 2.1. Subjects

Forty-three schizophrenia patients (22 men, 21 women) meeting the DSM-IV criteria for schizophrenia [10], in a stable phase of their illness and 43 healthy controls (22 men, 21 women) participated in the study. The groups were matched for age, handedness (Edinburgh Inventory) [11], and parental socioeconomic status (National Occupational Classification; NOC) [12] (Table 1).

All patients were reevaluated by experienced psychiatrists before being assigned to the research group (DSM-IV, criteria A-E); affective, schizoaffective and schizophreniform psychoses were excluded. Control participants were screened with the nonpatients edition of the Clinical Interview for DSM-IV (SCID) [13].

Symptom severity was rated according to the positive and negative syndrome scale (PANSS) [14]. Illness onset was defined as the date of the first psychiatric consultation. All the patients received at least one atypical antipsychotic (28 patients received one and 15 received two; clozapine: *n* = 20, mean dosage = 413,16 mgs ± 107,77 mgs; olanzapine: *n* = 12, mean dosage = 14.58 mgs ± 5.41 mgs; risperidone: *n* = 15, mean dosage = 3.14 ± 1.67 mgs; quetiapine: *n* = 9, mean dosage = 486,11 mgs ± 274.75 mgs; ziprasidone: *n* = 2, mean dosage = 166,65 mgs ± 47,14 mgs). All antipsychotic doses were calculated according to chlorpromazine equivalence [15].

General exclusion criteria included age below 18 or above 45 years, past or present neurological or Axis-I psychiatric disorder, alcoholism or drug abuse, noncompliance with testing procedures, abnormal uncorrected vision or any contraindication for MRI such as a cardiac pacemaker, an aneurysm clip, a metal prostheses, or cardiac valve replacement, the presence of metal in an eye or any part of the body, certain dental work, or claustrophobia.

In agreement with the Declaration of Helsinki, written informed consent was obtained prior to participation in the experiment. The ability of schizophrenia patients to give informed consent was established using the guidelines of the Canadian Psychiatric Association. The study was approved by the ethics committees of the Fernand-Seguin Research Center of the Louis-H Lafontaine Hospital and the Regroupement Neuroimagerie Québec.

### 2.2. Experimental Procedure

All participants underwent a functional magnetic resonance imaging (*fMRI*) scan during passively viewing blocks of emotionally positive, negative, and neutral pictures. The stimuli were selected from the International Affective Picture System (IAPS) [16] based on normative valence and arousal ratings, and images from each valence category (negative, positive, and neutral) were matched for content (e.g., people, animals, and landscapes). This task consisted of 48.5-second blocks of emotionally positive, negative, or neutral pictures and 16-second periods of rest separating the blocks from one another. Each block contained 10 images and was repeated 4 times. Each picture appeared for 3000 ms followed by a blank screen with a fixation point for an average of 1.75 s (ranging from 1 to 2.5 s and giving an average interstimulus interval (ISI) of 4.75 s). To assess the participants subjective emotional responses to the presented images, immediately at the end of the *fMRI* session, participants were represented with the images of each block and were asked to rate the block of images as whole on a scale ranging from 0 (absence of any emotional reaction) to 8 (strongest emotion ever felt in one's lifetime).

A blood sample of 10 mL was taken approximately 30 minutes prior to each scanning session to evaluate the levels of progesterone in all participants. The sample was immediately centrifuged and the serum separated. The samples were stored (−40 C) and later transported and analyzed at the laboratory of Maisonneuve-Rosemont Hospital. Serum levels of estradiol and progesterone were determined using the automated chemiluminescence assay system (SYNCHRON LXi 725, Beckman Coulter, USA). The analytical sensitivity was 0.08 ng/mL and dynamic range: 0.08–40.0 ng/mL.

### 2.3. *fMRI* Data Acquisition and Analysis

Blood-oxygenated dependent level (BOLD) signals were recorded using a single-shot, gradient-recalled echoplanar imaging sequence [repetition time (TR) = 3000 ms, echo time (TE) = 30 ms, flip angle = 90 degrees, matrix 64 × 64 voxels] on a MRI Siemens TRIO system at 3.0 Tesla, which is operational at the Functional Neuroimaging Unit at the University of Montreal Geriatric Institute.

The *fMRI* data were analyzed using statistical parametric mapping software (SPM5; Wellcome Department of Cognitive Neurology, London, UK) according to methods outlined by Friston and colleagues [17]. Functional images were realigned to the mean volume of each session to correct for artifacts due to subject motion, were spatially normalized into the standardized brain template (voxel size: 3.5 mm × 3.5 mm × 3.5 mm), and were spatially smoothed with a three-dimensional isotropic Gaussian kernel (12 mm FWHM) to improve the signaltonoise ratio.

Statistical analyses were carried out using a standard peak-detection approach and the general linear model implemented in SPM5 to identify the dynamic cerebral changes associated with emotional processing task. First, *fMRI* data of each participant was analyzed using a fixed-effects model to investigate individual brain activation maps and to contrast the brain activity associated with different contrasts. The fixed-effects analysis produced individual contrast images that were then used as raw data for the implementation of a random-effects model to investigate the pattern of activations during the different emotional contrasts (positive minus neutral and negative minus neutral) in each group (i.e., healthy men, healthy women, schizophrenia men, and schizophrenia women). We further examined potential differences between groups (healthy controls minus schizophrenia patients) as well as between groups within the same sex (e.g., activations in healthy women minus activations in schizophrenia women, etc.) using a two-sample *t*-test. Due to the strict character of the second-level analysis based on a random-effects model, the statistical maps were threshold at a level of *P* = 0.005 uncorrected for multiple comparisons.

To assess correlations between progesterone and brain function, second-level regression analyses were performed in SPM5. Progesterone levels were entered as covariates of interest. These were correlated with brain function during the processing of negative and positive images separately. The threshold level for statistical significance was set at a *P* = 0.001 corrected for multiple comparisons using the small volume correction (SVC) with the sphere volume function in SPM5 (radius = 12 mm). These correlations were done for healthy and schizophrenia men and women separately at the whole-brain level.

The demographic and clinical data were analyzed with the statistical package for the social sciences (SPSS), version 15.0. To examine ratings of emotional stimuli and recognition accuracy we conducted a repeated measures ANOVA with image type (i.e., NEG and POS) as a within-subject factor and diagnostic group and sex as between-subject factors. Where group or stimulus effects were detected the source of these effects was further investigated using post hoc *t*-tests.

## 3. Results

### 3.1. Stimulus Rating

ANOVA revealed no main effect or group or sex and no significant interaction between the two variables (Table 1).

### 3.2. *fMRI*—Between-Group Comparisons

Individuals with schizophrenia showed decreased activations in the left lingual gyrus (*z* = 3.28, *k* = 63, *P* = 0.001) relative to healthy subjects during the processing of positive emotions. No significant group differences were observed during negative emotion processing.

With regards to sex differences, schizophrenia women showed decreased brain activity in bilateral cuneus (*z* = 3.23, *k* = 493, BA = 18, *P* = 0.001), left lingual gyrus (*z* = 3.14, *k* = 493, BA = 19, *P* = 0.001), and right cerebellum (*z* = 3.04, *k* = 12, *P* = 0.001) relative to healthy women during the processing of positive emotion processing. No significant differences between healthy and schizophrenia women were observed during negative emotion processing. No significant differences were observed between healthy and schizophrenia men during the processing of positive or negative emotion.

### 3.3. *fMRI*—Correlations between Positive Emotion and Progesterone

**Figure 1:**
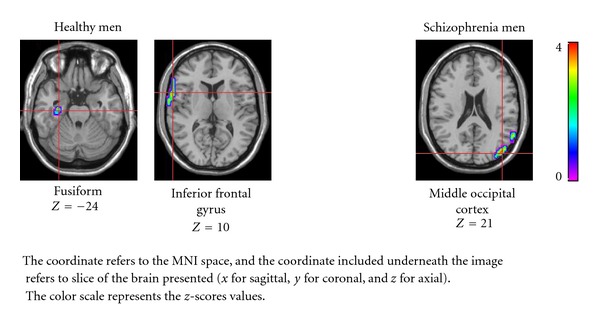
Correlation between progesterone levels and brain activations during processing of positive images.

Analysis in healthy men revealed a positive correlation between progesterone levels and brain activations in the left fusiform gyrus (*z* = 3.07, *k* = 8, BA = 36, *P* = 0.044) and a trend in the inferior fontal gyrus (*z* = 3.00, *k* = 58, BA = 44, *P* = 0.05) (Figure 1). In schizophrenia men, a positive correlation was observed in the middle occipital cortex (*z* = 3.16, *k* = 76, BA = 19, *P* = 0.01). No significant relationship was observed in either healthy or schizophrenia women.

### 3.4. *fMRI*—Correlations between Negative Emotion and Progesterone

**Figure 2:**
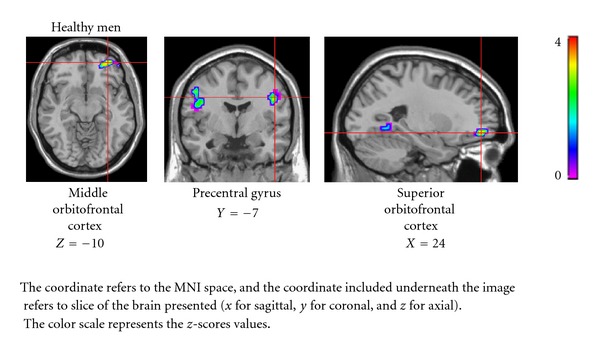
Correlation between progesterone levels and brain activations during processing of negative images.

In healthy men, there was a positive correlation between progesterone levels and brain activations in the middle orbitofrontal cortex (*z* = 3.25, *k* = 40, BA = 10, *P* = 0.034), superior orbitofrontal cortex (*z* = 3.12, *k* = 40, BA = 11, *P* = 0.041), and a trend was observed in the precentral gyrus (*z* = 2.99, *k* = 15, BA = 6, *P* = 0.064) (Figure 2). No significant relationship between brain function and progesterone levels was seen in healthy women, schizophrenia women, or schizophrenia men.

## 4. Conclusion

To our knowledge, this is the first investigation of progesterone's implication in neural circuitry underlying emotional processing in men and women with schizophrenia. First, however, we had to establish if there were any between-group differences in cerebral activations during viewing of emotional stimuli. The direct comparison between schizophrenia patients and healthy controls revealed only limited differences: relatively less activation in the left lingual gyrus in the patient group during positive condition and no group differences in the negative condition. When we separated the groups by sex, we found that it was women with schizophrenia who demonstrated decreased activity during the positive condition in the posterior cortex, bilateral cuneus, left lingual gyrus, and right cerebellum. Although these differentially activated regions are not considered to be the core centers of emotion processing, the occipital cortex has been frequently activated during visual emotional tasks [18], while activations in the cerebellum have been reported during the induction of feelings of sadness, anxiety [19, 20], happiness [21], in evoking romantic love [22], and in the “feeling” experience associated with sexual arousal [23].

The main finding of the present study, however, was that progesterone (studied in the past mostly in the context of the female reproductive function) appeared to be associated with the cerebral activations during emotion processing in men but not in women. Thus, during negative emotion processing, the activations in the middle and superior orbitofrontal cortex were significantly correlated with progesterone levels in healthy men only. Numerous neuroimaging studies have shown that the orbitofrontal cortex is implicated in the processing of negative stimuli [24–26], but the role of progesterone has not been addressed. In comparison, during the processing of positive emotion, progesterone levels were correlated with activations in the fusiform gyrus in healthy men and with the middle occipital cortex in male patients. As mentioned above, occipital cortex has been frequently activated during emotional task using visual stimuli. Also, it has been well established that emotional face processing is directly linked with fusiform gyrus activation [27]. Thus, if we assume an implication of progesterone in the overall emotion processing, the significant correlations between this steroid hormone and cerebral activations during affective task are not unexpected. What remains surprising, however, is that there was no association between progesterone and emotional processing in either healthy or schizophrenia women, despite progesterone's role in mood regulation of female subjects (in both human and animal studies) [4, 28, 29].

Animal studies have shown that progesterone receptors are present in several limbic and corticolimbic structures (traditionally associated with affect and emotion processing), including hypothalamus, thalamus, amygdala, hippocampus, prefrontal cortex, olfactory bulb and cerebellum [30–32]. Moreover, one postmortem study in women revealed concentrations of progesterone and its metabolites in the prefrontal, temporal, and parietal cortex, as well as in some subcortical structures including amygdala, hippocampus, caudate, putamen, thalamus, nucleus accumbens, substantia nigra, hypothalamus, and cerebellum [33]. Therefore, our findings of significant correlations between progesterone levels and brain activations in orbitofrontal cortex in male participants are somewhat consistent with those receptor binding and postmortem studies.

Several studies have shown that progesterone and its derivative, allopregnanolone, have a modulating effect on neurotransmitters systems involved in the regulation of emotion, such as serotonin and noradrenalin [34, 35]. Traditionally, progesterone has been investigated in the context of the female menstrual cycle, contraception, or breast cancer. However, our results show that, at least in the case of cerebral activations during emotional processing, this hormone appears to be more important for men than for women. In other words, although progesterone plays a primary role in the female reproductive function, it may be also important for the brain function in males.

Thus despite the fact that our principal motivation for the present study was to examine differences between schizophrenia patients and healthy controls, our most intriguing finding was the similar pattern of sex differences in both groups (i.e., progesterone had a significant association with cerebral activations in men but not women). It should be added that this relationship was more pronounced in healthy men than in schizophrenia men.

Although intriguing, this data is preliminary and warrants further investigation. For example, it is possible that with greater number of female participants we would also find a significant association between progesterone and brain activation during emotional processing, though the pattern of these results might differ. The findings emphasize the importance of including both sexes (and investigating them separately) in neuroendocrine and neuroimaging studies of schizophrenia and other psychiatric disorders that show sex differences in epidemiological, clinical, or neurobiological profile (e.g., major depression, autism, and ADHD).

## Figures and Tables

**Table 1:** Sociodemographic and clinical characteristics.

	Subjects with schizophrenia		Normal control subjects	
	Women (*N* = 21)	Men (*N* = 22)	Women (*N* = 21)	Men (*N* = 22)
Age (years) (mean and SD)	32.86 (6.56)	31.36 (7.36)	29.28 (9.27)	30.55 (7.81)
Parental SES (mean and SD)	2.54 (1.05)	2.79 (0.75)	2.08 (1.10)	2.43 (1.11)
Handedness, no. right (%) (mean and SD)	21 (86.15)	18 (60.47)	21 (65.39)	18 (54.36)
Age at onset (mean and SD)	24.29 (6.14)	20.38 (3.90)**		
Duration of Illness years (mean and SD)	8.14 (5.66)	10.95 (7.64)		
Chlorpromazine equivalents, mg (mean and SD)	467.06 (292.26)	693.18 (378.41)**		
PANSS positive (mean and SD)	19.32 (7.82)	18.18 (5.51)		
PANSS negative (mean and SD)	20.14 (8.69)	20.09 (5.45)		
PANSS general (mean and SD)	42.27 (12.72)	39.32 (6.02)		
Subjective rating positive (mean and SD)	4.75 (1.20)	4.95 (1.35)	5.06 (0.95)	4.38 (1.15)
Subjective rating negative (mean and SD)	5.14 (1.24)	5.36 (1.17)	5.75 (0.58)	5.04 (1.06)

**Significant *P* < 0.05.
